# Precision base-editing of the cryptic 3’ acceptor site to correct the RNA splicing defect of β^654^-thalassemia

**DOI:** 10.1186/s43556-026-00589-z

**Published:** 2026-09-30

**Authors:** Dan Lu, Xiuli Gong, Xinbing Guo, Qin Cai, Wenxiu Li, Yanwen Chen, Dali Li, Fanyi Zeng

**Affiliations:** 1 Shanghai Institute of Medical Genetics, Shanghai Children’s Hospital, Shanghai Jiao Tong University School of Medicine, Shanghai, 200040 China; 2 Department of Histo-Embryology, Genetics and Developmental Biology, Shanghai Jiao Tong University School of Medicine, Shanghai, 200025 China; 3 NHC Key Laboratory of Medical Embryogenesis and Developmental Molecular Biology & Shanghai Key Laboratory of Embryo and Reproduction Engineering, Shanghai, 200040 China; 4 Shanghai Frontiers Science Center of Genome Editing and Cell Therapy, Shanghai Key Laboratory of Regulatory Biology, Institute of Biomedical Sciences and School of Life Sciences, East China Normal University, Shanghai, 200241 China; 5 School of Pharmacy, Macau University of Science and Technology, Macau, China

**Keywords:** Base editor, β^654^-thalassemia, Gene therapy, RNA splicing

## Abstract

β-Thalassemia is one of the most common inherited disorders worldwide and is caused by mutations affecting β-globin production. β^654^ mutation (IVS2-654, C > T) is one of the most frequently occurring β-thalassemia alleles in Han Chinese population, which activates a cryptic 3′ splice site and leads to aberrant RNA splicing. Previous studies demonstrated that direct base editing of the IVS2-654 mutation is challenging because suitable single-guide RNAs (sgRNAs) cannot effectively target this site. Here we investigated an alternative therapeutic strategy by targeting the mutation-activated cryptic 3′ splice acceptor site, rather than the disease-causing mutation itself. We first introduced base substitutions into the cryptic splice acceptor site in a β^654^-thalassemia mouse model using CRISPR-Cas9-mediated homology-directed repair, which restored normal RNA splicing and validated the therapeutic rationale of this approach. We then generated base-edited β^654^ mice by microinjecting Td-CBEmax mRNA together with sgRNAs targeting the cryptic splice acceptor site into one-cell embryos. Base editing was successfully achieved in 78% of live-born β^654^ mice, of which 86% produced correctly spliced β-globin transcripts. Restoration of normal RNA splicing was accompanied by marked improvement of hematological parameters and tissue pathology in most base-edited founder mice and their offspring compared with non-edited β^654^ mice. Together, these findings demonstrate that mutation-activated cryptic splice sites are therapeutically actionable targets for precision base editing and provide proof of concept for a precise and effective strategy to correct aberrant RNA splicing in β^654^-thalassemia.

**Supplementary Information:**

The online version contains supplementary material available at https://doi.org/10.1186/s43556-026-00589-z.

## Introduction

β-Thalassemia is one of the most common inherited hematological disorders worldwide and is characterized by reduced or absent β-globin synthesis. It is caused by pathogenic variants in the β-globin gene (*HBB*) that impair the production of functional β-globin chains [1]. Current treatment options, including regular blood transfusions and iron chelation therapy, alleviate disease symptoms but do not correct the underlying genetic defect. Although allogeneic hematopoietic stem cell transplantation offers a potential cure, its application is limited by donor availability and the risks of graft-versus-host disease, immune rejection, and transplantation-related complications [2, 3]. These limitations have stimulated the development of gene-editing strategies aimed at restoring endogenous *HBB* function.

More than 350 pathogenic variants have been identified in *HBB* [4, 5], among which splicing-disrupting mutations account for a major proportion [6]. Many such variants activate silent cryptic splice sites, which compete with canonical splicing signals, shift open reading frames, and generate nonfunctional globin transcripts. The IVS2-654 (C > T) mutation, one of the most prevalent β-thalassemia mutations in the Han Chinese population, exemplifies this mechanism [7]. It creates an abnormal 5′ splice donor site and activates a downstream cryptic 3′ splice acceptor site, thereby severely suppressing mature β-globin synthesis [8, 9]. Disrupting this mutation-induced cryptic splice site therefore represents a promising therapeutic strategy for IVS2-654-type β-thalassemia.

Several gene-based therapeutic approaches for β-thalassemia have advanced into clinical development. Mutation-independent strategies, including γ-globin induction and lentiviral-mediated *HBB* gene addition, have demonstrated encouraging clinical efficacy [10–13]. In parallel, genome-editing technologies such as CRISPR-Cas9 and Cas12a have enabled precise modification of disease-associated loci through non-homologous end joining or homology-directed repair [14, 15]. Previous studies, including our own work, have demonstrated that the aberrant splicing caused by the IVS2-654 mutation can be corrected by targeting aberrant splice elements within intron 2 of *HBB*. CRISPR-Cas9-mediated deletion of the mutation-generated 5′ splice donor site and/or the activated cryptic 3′ splice acceptor site restored normal β-globin RNA splicing and ameliorated β-thalassemia phenotypes in β^654^ mice [16–20]. HDR-mediated single-base alteration at this cryptic site further verified its therapeutic value [21]. Together, these studies established the functional importance of the mutation-activated cryptic splice site and validated it as a therapeutic target. However, nuclease-based approaches rely on DNA double-strand breaks and may generate heterogeneous deletions, indels, chromosomal rearrangements, or variable repair outcomes, which may limit their therapeutic predictability [22–24]. Therefore, a more precise, DSB-free strategy for modifying this validated cryptic splice-site target remains needed.

Base editors enable defined single-nucleotide conversions without generating DNA double-strand breaks and therefore provide an attractive approach for precise modification of pathogenic sequence elements [25]. Existing base-editing strategies for β-thalassemia generally fall into two categories. The first directly targets disease-causing mutations when suitable protospacer adjacent motifs and compatible editing windows are available. Successful correction has been reported for several β-thalassemia-associated mutations, including *HBB*−28 (A > G), as well as mutations responsible for sickle cell disease [26, 27]. However, direct correction is highly dependent on target accessibility, making the IVS2-654 mutation difficult to edit using currently available base editors [28, 29]. The second strategy increases fetal hemoglobin expression by editing regulatory genes or elements [4]. Although this approach can improve clinical manifestations, it does not restore endogenous β-globin expression or correct the underlying splicing defect. For β^654^-thalassemia, it remains unclear whether the validated cryptic splice acceptor site can be precisely modified by base editing while preserving the native genomic context.

In the present study, we aimed to establish a DSB-free base-editing platform to correct the mutation-activated cryptic 3′ splice acceptor site in IVS2-654 β-thalassemia. We first used CRISPR-Cas9-mediated homology-directed repair to validate that defined nucleotide substitutions at this site can restore normal β-globin RNA splicing. We then employed the cytosine base editor Td-CBEmax, chosen for its narrow editing window and high single-base precision, to perform DSB-free targeted base conversion at the cryptic splice site [30]. Our results demonstrate that Td-CBEmax-mediated editing restores normal β-globin RNA splicing, recovers endogenous β-globin expression, ameliorates hematological and histopathological phenotypes, and exhibits a favorable in vivo specificity profile. These findings advance cryptic splice-site correction from nuclease-based target validation toward a more precise and predictable base-editing framework.

## Results

### The cryptic 3′ splice acceptor site serves as an alternative therapeutic target for correcting the RNA splicing defect in β^654^ mice

**Fig. 1:**
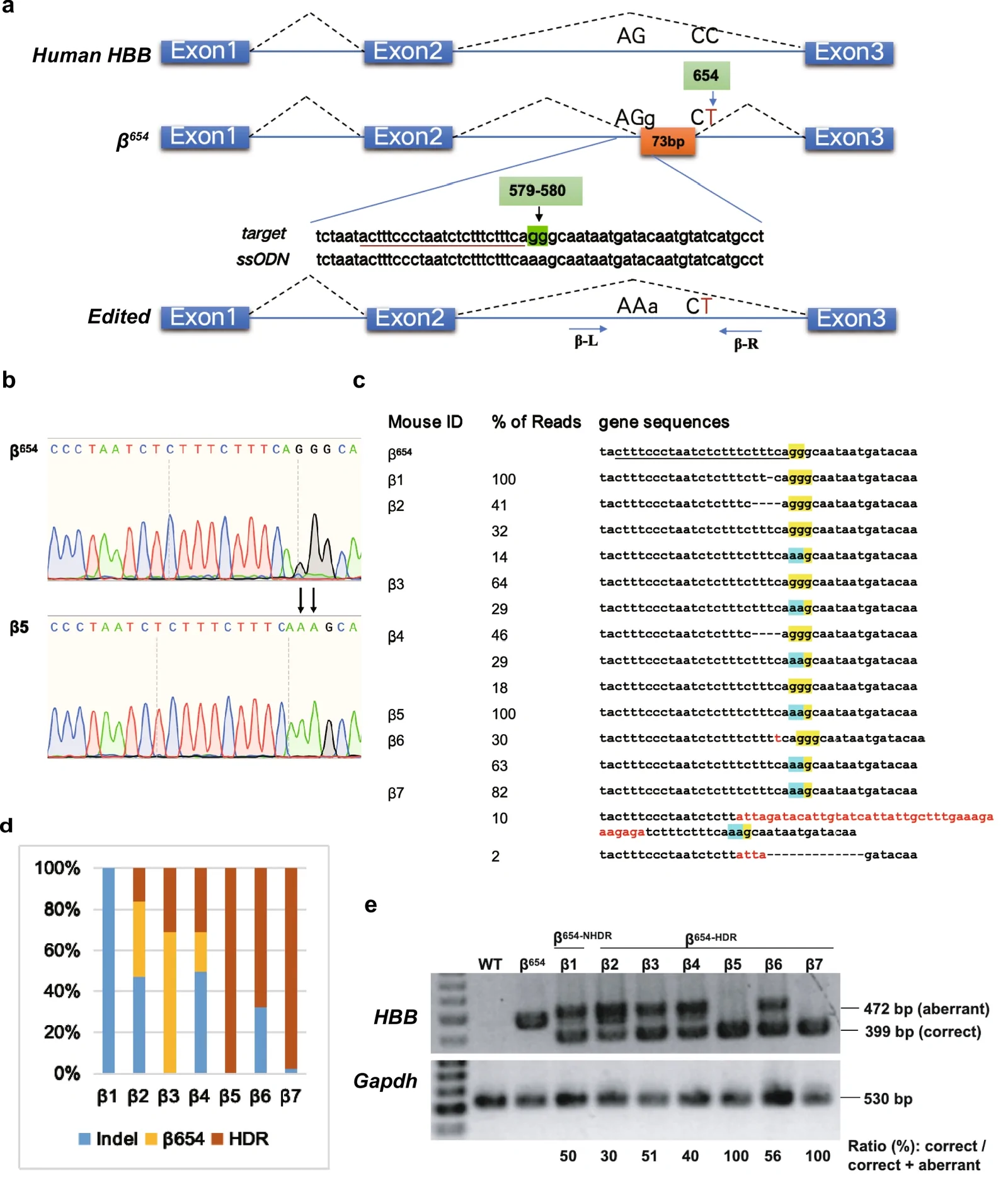
Generation of β^654−HDR^ mice with human β-globin splicing correction. **a** Schematic illustration of the aberrant splicing process of human β^654^-globin pre-mRNA and the molecular correction strategy using CRISPR-Cas9-mediated homology-directed repair. Underlined sequences indicate the sgRNA targeting region upstream of IVS2-579_580. Blue arrows denote PCR primer positions. **b** Representative Sanger sequencing chromatograms of β^654^ mice and F0 mice injected with Cas9 mRNA and ssODN. **c** Genotyping of representative F0 mice following HDR. Allele frequencies were determined by deep sequencing. Yellow shading indicates PAM sequences; blue bases indicate substitutions; red bases indicate insertions; “–” indicates deletions. **d** Frequency of HDR-mediated modification in F0 mice. Indel, β^654^ alleles with insertion or deletion mutations; HDR, β^654^ alleles with homology-directed repair modifications; β^654^, β^654^ alleles. **e** RT-PCR analysis of human β-globin mRNA splicing. WT, wild-type; β^654^, β^654^ mouse; β1, β^654−NHDR^; β2–7, β^654−HDR^ mice. WT lane represents wild-type mice lacking the human β-globin transgene, and therefore no human β-globin RT-PCR product is detected

The IVS2-654 mutation generates an aberrant 5′ splice donor site and activates a cryptic 3′ splice acceptor site, leading to abnormal β-globin pre-mRNA splicing. Because activation of this cryptic splice site is essential for the aberrant splicing process, we first investigated whether precise modification of the cryptic 3′ splice acceptor site could restore normal RNA splicing. To test this hypothesis, CRISPR-Cas9-mediated homology-directed repair (HDR) was employed to introduce base substitutions at the cryptic 3′ splice acceptor site. A 59-bp single-stranded oligodeoxynucleotide (ssODN) carrying IVS2-579_580 (GG > AA) substitutions was designed as the donor template. These substitutions disrupted the protospacer adjacent motif (PAM), thereby preventing repeated Cas9 cleavage following HDR-mediated repair and enhancing editing specificity (Fig. 1a). Two candidate sgRNAs (sgRNA-1 and sgRNA-2; Table S1) targeting sequences adjacent to the IVS2-579 locus were initially evaluated by bioinformatic prediction. Because sgRNA-1 exhibited a lower predicted off-target risk while remaining proximal to the target site, it was selected for subsequent HDR experiments.

Following microinjection of sgRNA-1, Cas9 mRNA, and the ssODN donor into fertilized zygotes, six gene-edited β^654^ founder mice carrying the intended IVS2-579_580 (GG > AA) substitution were obtained (Fig. 1b–d). RT-PCR analysis demonstrated that correctly spliced β-globin transcripts were detected in all mice carrying the HDR-modified allele (Fig. 1e). Together, these results demonstrate that precise modification of the cryptic 3′ splice acceptor site is sufficient to restore normal β-globin RNA splicing in β^654^ mice, thereby establishing this mutation-activated splicing element as a feasible therapeutic target for subsequent base-editing studies.

### Base editor Td-CBEmax efficiently modifies the cryptic 3′ splice acceptor site in HEK293T cells

**Fig. 2:**
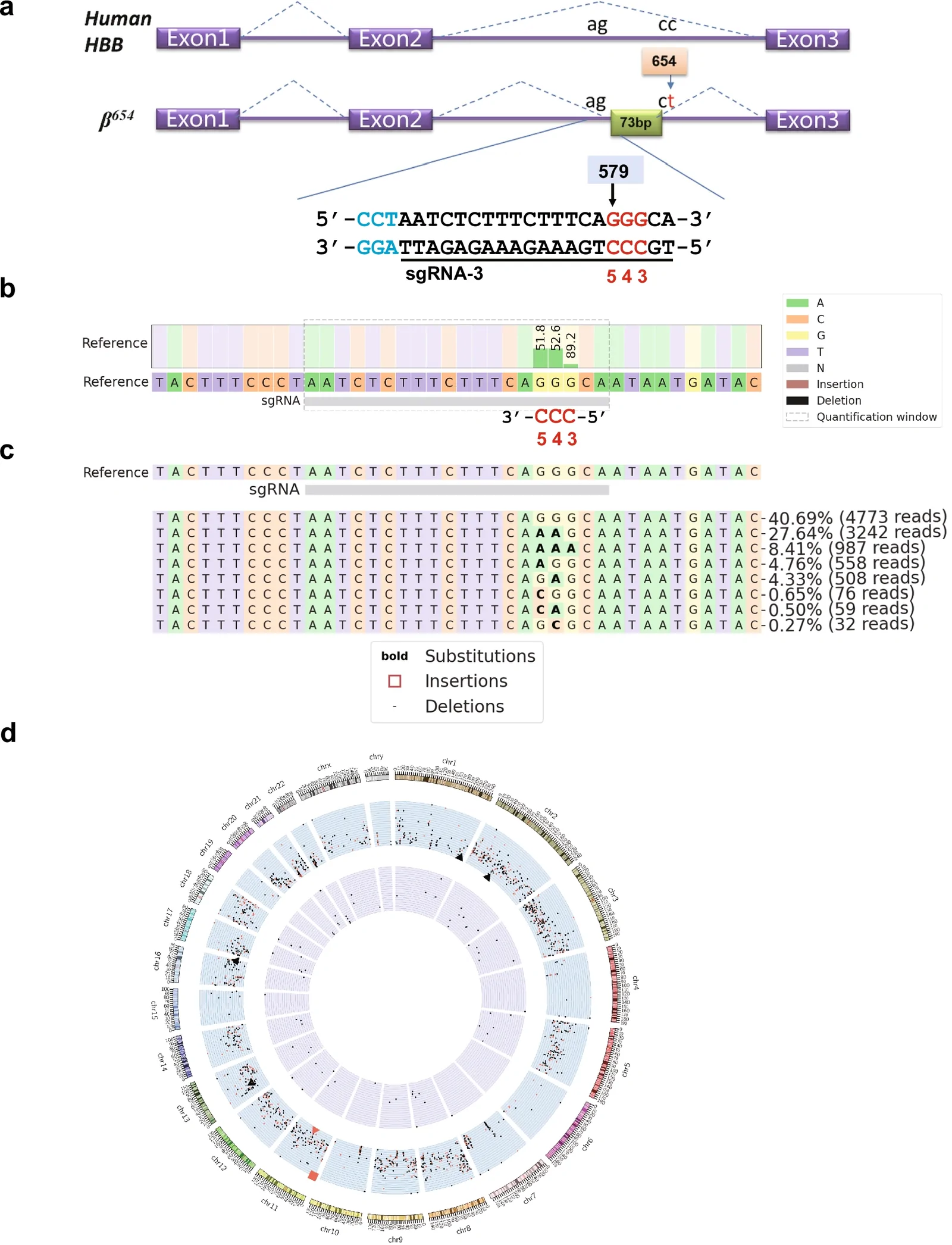
sgRNA design and base-editing on- and off-target profiles in HEK293T cells. **a** Schematic of the target sequence in intron 2 of the human β-globin gene harboring the IVS2-654 mutation. The sgRNA target sequence is underlined, and the predicted editing window corresponding to IVS2-579_581 is indicated. Protospacer adjacent motifs are highlighted. **b** Nucleotide distribution surrounding the sgRNA target site following Td-CBEmax editing. **c** Distribution of edited alleles at the target locus as determined by deep sequencing. Nucleotides are color-coded (A, green; C, red; G, yellow; T, purple). Substituted bases are shown in bold. **d** Genome-wide distribution of potential off-target events identified by whole-genome sequencing. Squares indicate on-target sites, and triangles indicate sgRNA-dependent off-target sites. Red represents C to T or G to A; black represents other base changers

Having established that modification of the cryptic 3′ splice acceptor site is sufficient to restore normal RNA splicing, we next investigated whether this site could be efficiently edited using the cytosine base editor Td-CBEmax. Based on the predicted on-target activity and off-target profiles generated by CRISPOR [31], two candidate sgRNAs (sgRNA-3 and sgRNA-4) were selected because their predicted editing windows encompassed the cryptic 3′ splice acceptor site (Fig. 2a, Fig. S1a, and Table S1). Each sgRNA was co-transfected individually with the Td-CBEmax expression plasmid into HEK293T cells to evaluate editing efficiency. Targeted deep sequencing demonstrated combined C•G-to-T•A editing frequencies of 45.55% and 30.09% for sgRNA-3 and sgRNA-4, respectively (Fig. 2b, c and Fig. S1b, c). Because sgRNA-3 exhibited substantially higher editing efficiency, it was selected for all subsequent experiments. The predicted editing window of sgRNA-3 spanned positions C5–C3, corresponding to IVS2 positions 579–581 (G5–G3) (Fig. 2a). In addition to the intended C•G-to-T•A conversion, a low frequency of unintended G-to-C editing (~ 1%) was detected at the G5 position (Fig. 2c).

The specificity of Td-CBEmax-mediated editing was first evaluated by targeted deep sequencing of the ten highest-ranked predicted off-target sites. Only one candidate site (OT9; hg19 chr4:170,395,548–170395570) exhibited significantly higher editing than the untreated control. Editing efficiencies at the predicted target cytosines (C2, C4, and C5) were 0.3%, 1.3%, and 2.4%, respectively (Table S2 and Fig. S2). Importantly, this site is located within an intergenic region lacking annotated coding sequences, known regulatory elements, or conserved genomic features (Table S2 and Fig. S2). To further evaluate genome-wide editing specificity, whole-genome sequencing (WGS) was performed on edited and untreated HEK293T cells. Base editing was detected exclusively at the intended on-target locus, and no insertions or deletions were identified at the predicted off-target sites (Fig. 2d). Together, these results demonstrate that Td-CBEmax mediates efficient and highly specific base editing of the cryptic 3′ splice acceptor site in HEK293T cells, supporting its application for subsequent in vivo studies.

### Td-CBEmax efficiently edits the cryptic 3′ splice acceptor site in β^654^ mice

**Fig. 3:**
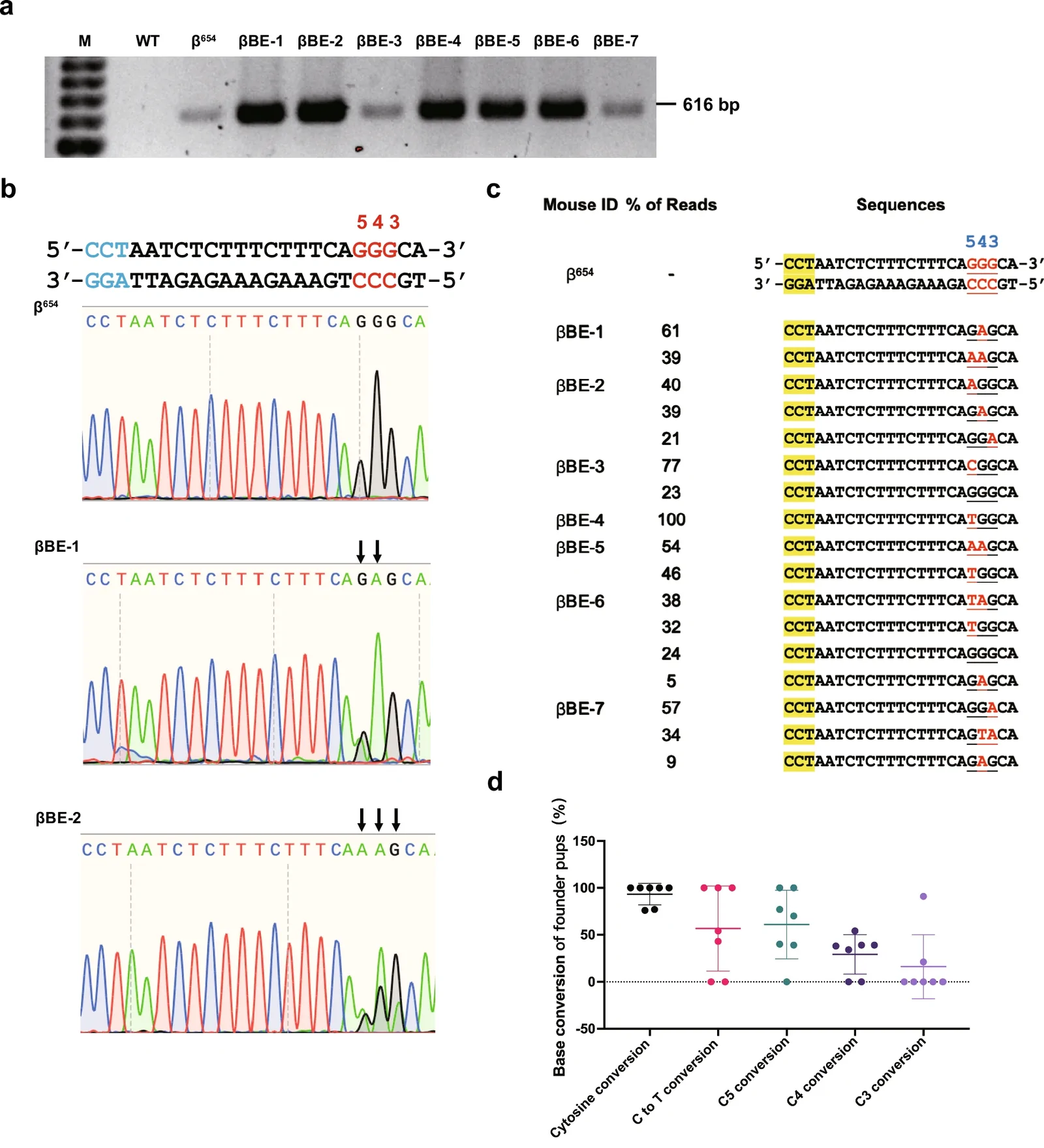
Identification and genotyping of β^654−BE^ mice. **a** PCR-based identification of β^654^ mice. WT: wild-type mouse; β^654^: β^654^ mouse; βBE-1 to βBE-7: β^654−BE^ mice. **b** Representative Sanger sequencing chromatograms from β^654^ mice and β^654−BE^ founder mice (βBE-1 and βBE-2) injected with Td-CBEmax mRNA. **c** Genotyping of representative β^654−BE^ founder mice based on deep sequencing. The frequencies of edited alleles are shown. Yellow: PAMs. Red: substituted bases. **d** Cytosine base conversion frequencies in β^654−BE^ founder mice (*n* = 7)

Having demonstrated efficient and specific base editing of the cryptic 3′ splice acceptor site in HEK293T cells, we next evaluated whether Td-CBEmax could mediate efficient editing in vivo in the β^654^-thalassemia mouse model. To generate base-edited mice, sgRNA-3 and Td-CBEmax mRNA were microinjected into fertilized zygotes obtained from β^654^ × WT matings. A total of 47 pups survived to weaning age, among which 19% (9/47) carried the β^654^ allele. Sanger sequencing demonstrated that 78% (7/9) of these β^654^ mice harbored detectable base editing at the target locus (Fig. 3a, b and Fig. S3). These mice were subsequently designated as β^654−BE^ mice.

Targeted deep sequencing further demonstrated that more than 70% of β^654^ alleles carried cytosine base conversions in all β^654−BE^ mice. Among the β^654−BE^ mice, 71% (5/7) harbored β-globin alleles with the intended G > A substitution. In addition, 29% (2/7) of the β^654−BE^ mice carried alleles with G > T or G > C substitutions (Fig. 3c, d). Furthermore, 86% (6/7) of the β^654−BE^ mice carried cytosine conversion alleles at the IVS2-579 site, which accounted for more than 39% of total alleles detected (Fig. 3c). Despite delivery of the editing components at the one-cell stage, editing events occurred at the two-cell stage or later in some embryos, resulting in mosaic genotypes among founder animals. The frequency of base-edited alleles in individual β^654−BE^ mice ranged from 5 to 100%. Together, these results demonstrate that Td-CBEmax efficiently edits the mutation-activated cryptic 3′ splice acceptor site in β^654^ mice with high editing efficiency, providing a robust foundation for subsequent evaluation of RNA splicing correction and phenotypic rescue.

### Base editing restores normal β-globin RNA splicing and endogenous β-globin expression in β^654^ mice

**Fig. 4:**
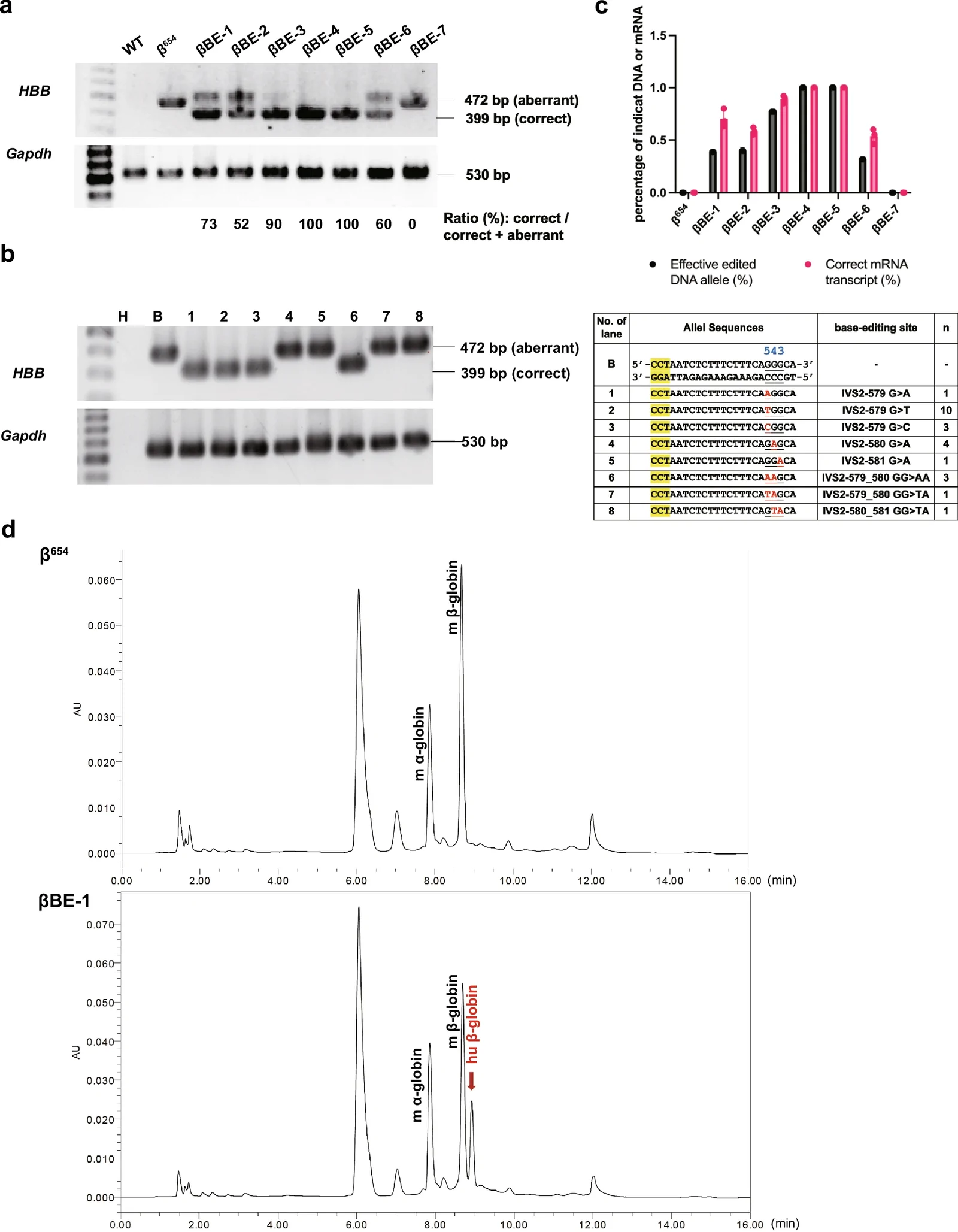
Correction of the RNA splicing defect in β^654−BE^ mice. **a** RT-PCR analysis of human β-globin mRNA splicing in WT, β^654^ and β^654−BE^ mice. **b** RT-PCR analysis of human β-globin mRNA splicing in the F1 generation of β^654−BE^ mice. H: H_2_O. **c** Relationship between the frequency of effective base-edited alleles and the proportion of correctly spliced human β-globin transcripts. **d** Representative high-performance liquid chromatography chromatograms showing globin chain composition. Peaks corresponding to human β-globin (red arrows) are indicated. hu β-globin: human β-globin, m α-globin: mouse α-globin, m βmajor: mouse β major globin

Having demonstrated efficient base editing of the mutation-activated cryptic 3′ splice acceptor site in β^654^ mice, we next investigated whether these nucleotide substitutions restored normal β-globin RNA splicing. RT-PCR analysis of human β-globin transcripts detected correctly spliced β-globin mRNA in six of the seven β^654−BE^ founder mice, whereas only aberrantly spliced transcripts were detected in untreated β^654^ mice (Fig. 4a). Subsequent Sanger sequencing of the RT-PCR amplicons further validated that the 399 bp band corresponded to correctly spliced β-globin transcripts, and the 472 bp fragment originated from aberrantly spliced products (Fig. S4). The proportion of correctly spliced transcripts ranged from 52 to 100% among these animals. Five of the six mice exhibiting corrected splicing carried multiple genotypes, consistent with mosaic base editing. To determine whether correction of RNA splicing was transmitted through the germline, β^654−BE^ founder mice were crossed with wild-type mice, and β-globin transcript splicing was analyzed in the resulting F1 offspring. Correctly spliced β-globin transcripts were detected in F1 mice carrying the edited allele (Fig. 4b).

To further evaluate the relationship between specific genotypes and splicing outcomes, transcriptional patterns were analyzed in the F1 generation following germline transmission and allele segregation. Among the eight genotypes identified, four were associated with restoration of normal splicing: IVS2-579 G > A, IVS2-579 G > T, IVS2-579 G > C, and IVS2-579_580 GG > AA. The remaining genotypes did not effectively disrupt recognition of the cryptic 3′ splice acceptor site (Table S3; Fig. 4b). The proportion of correctly spliced transcripts increased with the frequency of effective base-edited alleles in β^654−BE^ mice (Fig. 4c). HPLC showed the presence of human β-globin in five β^654−BE^ mice, consistent with the RT-PCR results (Fig. 4d and Fig. S5). Together, these results demonstrate that precise base editing of the mutation-activated cryptic 3′ splice acceptor site restores normal β-globin RNA splicing and endogenous β-globin expression in β^654^ mice.

### Base editing ameliorates the hematological abnormalities of β^654^-thalassemia mice

**Fig. 5:**
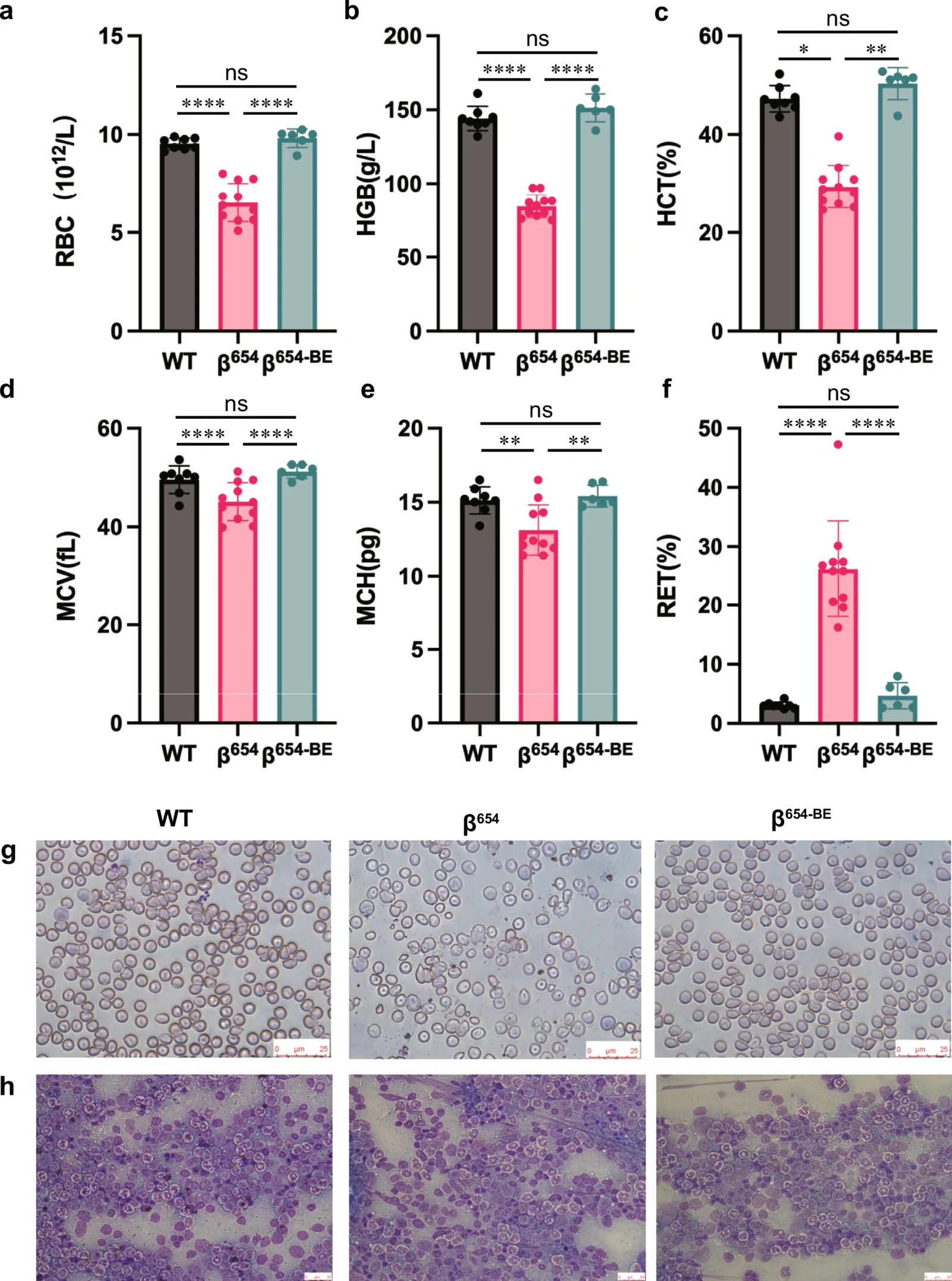
Hematological and cellular phenotypes in β^654−BE^ mice. **a**–**f** Hematological parameters of peripheral blood, including red blood cell count, hemoglobin concentration, hematocrit, mean corpuscular volume, mean corpuscular hemoglobin, and reticulocyte percentage. Data are presented as mean ± standard deviation. *P < 0.05, ** *P* < 0.01, **** *P* < 0.0001. **g** Representative Wright–Giemsa–stained peripheral blood smears from WT, β^654^, and β^654−BE^ mice. Red arrow: poikilocytosis; Black arrow: target cells in β^654^ mice. **h** Wright–Giemsa–stained bone marrow smears

Anemia is a primary clinical manifestation of insufficient β-globin synthesis. Having demonstrated restoration of normal β-globin RNA splicing and human β-globin expression, we next examined whether base editing improved the hematological abnormalities of β^654^-thalassemia mice. Compared with untreated β^654^ mice, β^654−BE^ mice exhibited significantly increased red blood cell counts (*P* < 0.01) and hemoglobin concentrations, with mean hemoglobin levels increasing from 84.6 g/L in β^654^ mice to 151.3 g/L in β^654−BE^ mice. Hematocrit, mean corpuscular volume, and mean corpuscular hemoglobin values were also significantly increased in β^654−BE^ mice compared with β^654^ mice (*P* < 0.001). In addition, reticulocyte counts were markedly reduced, decreasing from 26.2% in β^654^ mice to 4.7% in β^654−BE^ mice (Fig. 5a–f; Table S4). Peripheral blood smear analysis revealed marked anisocytosis, poikilocytosis, and red blood cell fragmentation in β^654^ mice, whereas these abnormalities were absent or substantially reduced in β^654−BE^ mice (Fig. 5g). Bone marrow smears from β^654^ mice showed abnormal proliferation and increased numbers of nucleated erythroid precursors, while these features were reduced in β^654−BE^ mice (Fig. 5h). Together, these results demonstrate that correction of the β-globin RNA splicing defect by base editing substantially alleviates the hematological abnormalities of β^654^-thalassemia mice.

### Base editing ameliorates splenomegaly and histopathological abnormalities in β^654−BE^ mice

**Fig. 6:**
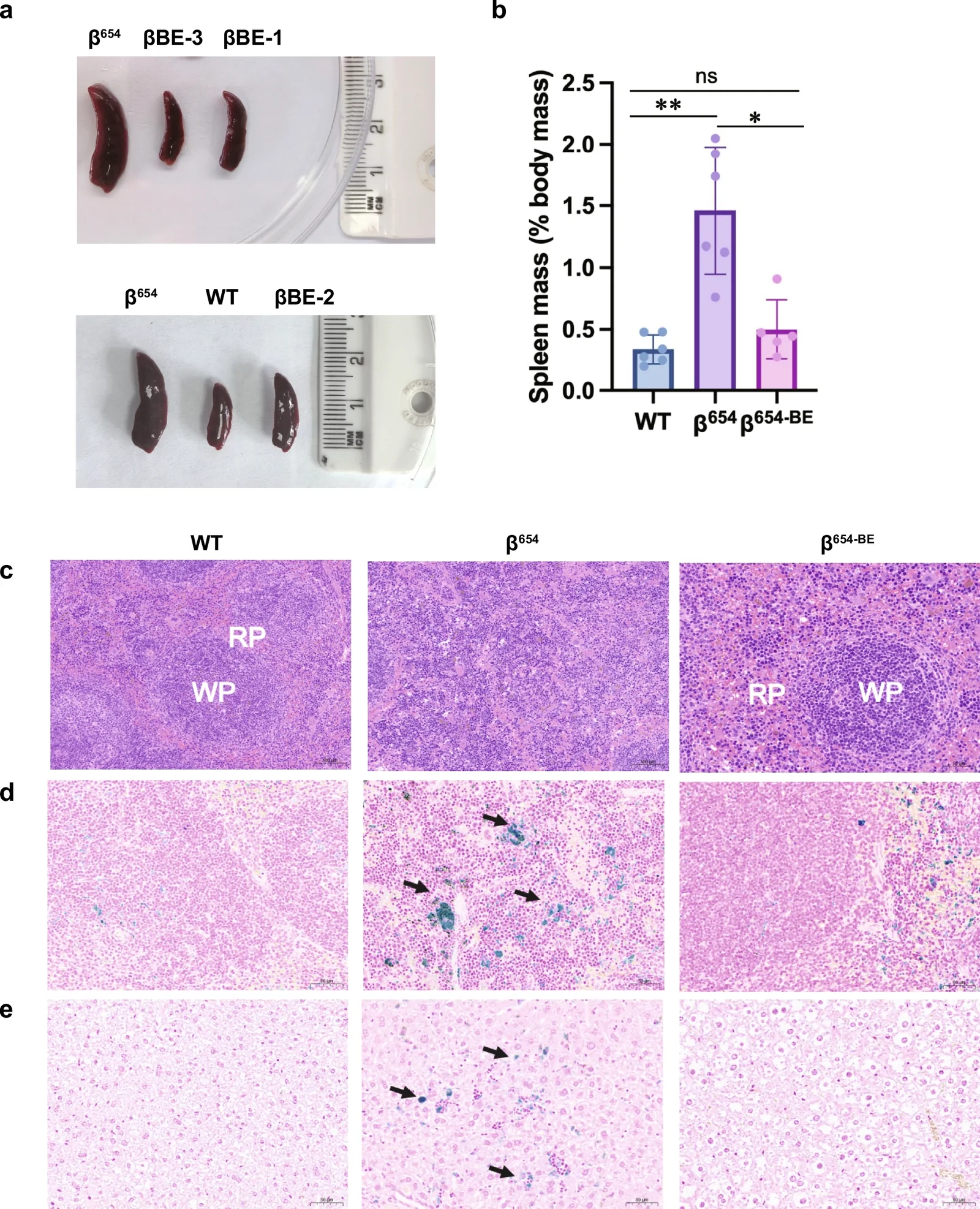
Splenomegaly and histopathological changes in β^654−BE^ mice. **a** Representative spleen images from WT, β^654^, and β^654−BE^ mice. **b** Spleen coefficient expressed as spleen mass relative to body mass. Data are presented as mean ± standard deviation. WT& β^654^, *n* = 6; β^654−BE^ *n* = 5. ** *P* < 0.01, ns: not significant. **c** Hematoxylin–eosin–stained spleen sections. RP, red pulp; WP, white pulp. **d** Prussian blue–stained spleen sections showing iron deposition. **e** Prussian blue–stained liver sections showing iron accumulation

Following the improvement of hematological parameters, we next evaluated whether base editing also ameliorated the pathological manifestations associated with β^654^-thalassemia. Gross examination demonstrated marked splenomegaly in untreated β^654^ mice, whereas spleen size was substantially reduced in β^654−BE^ mice (Fig. 6a). Quantitative analysis further showed that the spleen coefficient was significantly decreased in β^654−BE^ mice compared with untreated β^654^ mice (Fig. 6b).

Histological examination revealed pronounced pathological abnormalities in the spleen and liver of untreated β^654^ mice, including disrupted splenic architecture and extensive iron deposition. In contrast, β^654−BE^ mice exhibited substantial restoration of splenic tissue morphology, with clearer distinction between red pulp and white pulp, together with markedly reduced iron accumulation in spleen and liver tissues, as demonstrated by hematoxylin and eosin staining and Prussian blue staining (Fig. 6c–e). Together, these results demonstrate that correction of the β-globin RNA splicing defect by base editing is accompanied by significant amelioration of splenomegaly and tissue pathology associated with β^654^-thalassemia.

### Therapeutic effects of base editing are transmitted to the offspring of β^654−BE^ mice

To evaluate whether the therapeutic effects of base editing were transmitted to subsequent generations, β^654−BE^ mice were crossed with WT mice, and survival and hematological parameters were analyzed in the resulting F1 offspring. The survival rate of β^654^ mice in the F1 generation derived from β^654−BE^ parents was 37.5%, compared with 29.03% in control β^654^ mice generated by β^654^ × WT matings (Table 1). Hematological analysis of F1 β^654−BE^ offspring showed improvement in red blood cell indices relative to untreated β^654^ mice. No significant differences were observed in red blood cell count, hemoglobin concentration, or reticulocyte percentage between F1 β^654−BE^ mice and WT mice (Table S4). All F1 β^654−BE^ mice appeared healthy, and no pathological abnormalities associated with modification of the *HBB* locus were observed during more than one year of follow-up. Together, these results indicate that the therapeutic effects of base editing can be transmitted to the next generation and remain stable in offspring carrying the edited allele.

**Table 1:** The survival rate of β^654^ mice by gene editing

Mouse	Heterozygous β^654^	Total	β^654^ Survival Rate (%)
β^654−BE^ F1	18	48	37.5^a^
controls	45	155	29.03

F1 was produced by crossing β^654−BE^ with WT mice. Controls were produced by mating β^654^ with WT. ^a^*P* < 0.05

### Whole-genome sequencing supports the in vivo specificity of Td-CBEmax editing in β^654−BE^ mice

To characterize potential off-target alterations induced by Td-CBEmax editing in vivo, WGS was performed using spleen tissues from six β^654−BE^ mice and four untreated β^654^ control mice. Raw sequencing reads were aligned to the Mus musculus reference genome to identify single-nucleotide variants and small insertions/deletions. No statistically significant differences in the average numbers of genome-wide single-nucleotide variants or small insertions/deletions were observed between β^654−BE^ mice and untreated β^654^ control mice (Fig. S6 and Table S5). To further evaluate sgRNA-associated off-target events, the entire mouse reference genome was screened for sequences homologous to the sgRNA, allowing up to five base mismatches. A total of 650 candidate off-target intervals were identified (Table S6). For each candidate site, sequences spanning 100 bp upstream and downstream were examined for single-nucleotide variants and insertions/deletions. Among all analyzed β^654−BE^ mice, only one mouse, βBE1, harbored five candidate sgRNA-matched off-target variants, including one single-nucleotide variant and four insertions/deletions (Table S7). All five variants were located within intergenic regions. In addition, this mouse exhibited normal physical condition, with no detectable impairment of physiological function. Together, these results indicate that Td-CBEmax-mediated editing of the mutation-activated cryptic 3′ splice acceptor site exhibits a favorable in vivo genome-wide specificity profile in β^654−BE^ mice.

**Fig. 7:**
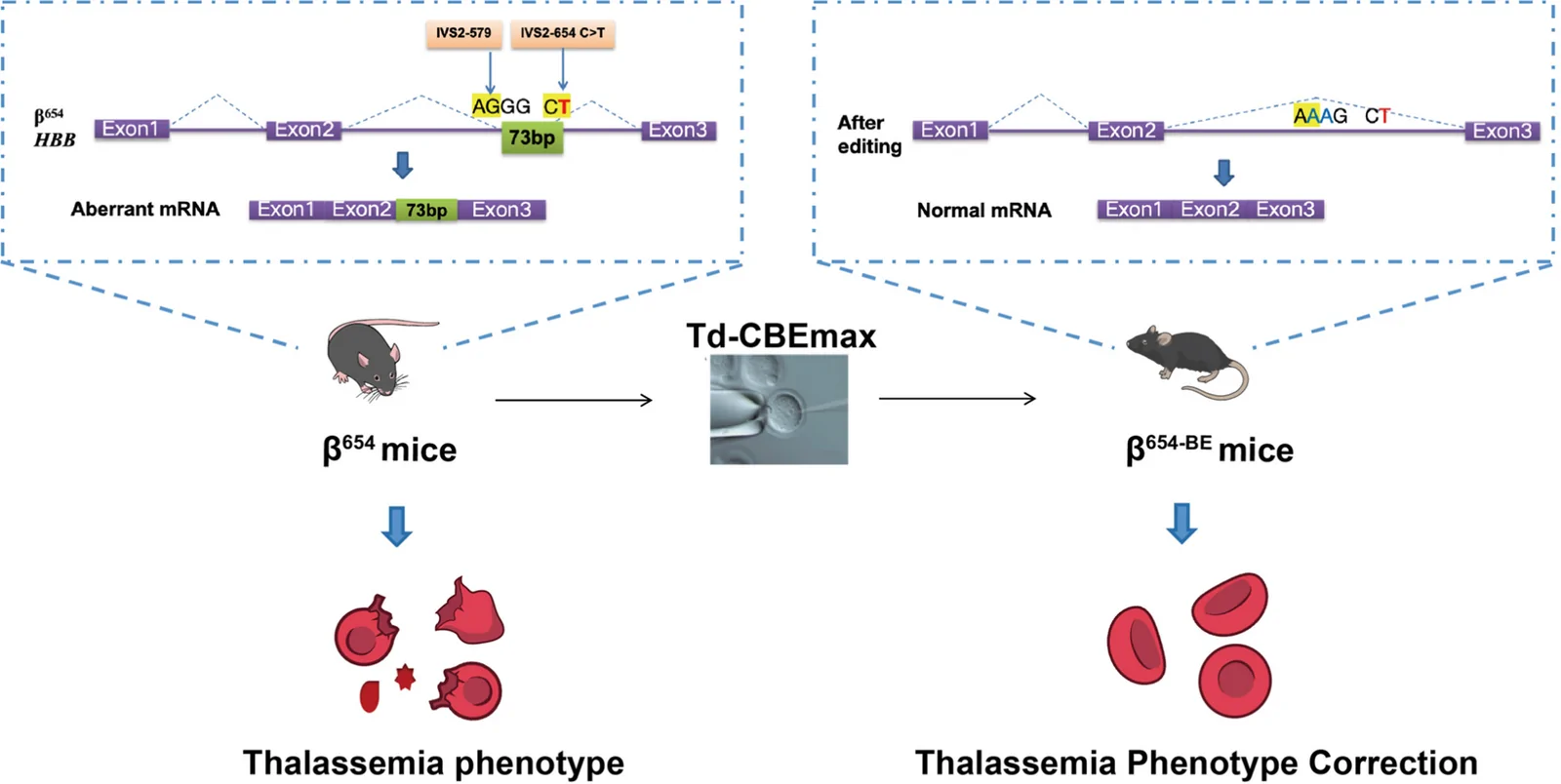
Schematic summary illustrating the therapeutic correction of β^6^^54^-thalassemia via Td-CBEmax base editing. Left panel: The pathogenic IVS2-654 C > T mutation activates a cryptic 3′ splice acceptor site accompanied by the IVS2-579 auxiliary motif, leading to retention of a 73 bp intronic segment in mature *HBB* mRNA. Aberrant globin transcripts are associated with deformed and fragmented red blood cells and typical severe thalassemia phenotypes in unedited β⁶^54^ model mice. Right panel: Delivery of the Td-CBEmax base editor remodels the abnormal splice motif (AGGG to AAAG), abolishes cryptic splice-site recognition, and restores canonical splicing of full-length *HBB* mRNA. After base editing, β⁶^54−BE^ mice exhibit improved erythrocyte morphology and substantial rescue of thalassemia-related hematological disease phenotypes

In summary, this study demonstrates that precise Td-CBEmax-mediated base editing of the mutation-activated cryptic 3′ splice acceptor site restores normal β-globin RNA splicing and substantially rescues disease-associated phenotypes in a β^654^-thalassemia mouse model. Building on previous nuclease-based validation of this pathogenic splicing element, the present work advances cryptic splice-site correction into a defined, DSB-free base-editing framework. By targeting the molecular event responsible for aberrant RNA processing rather than directly correcting the disease-causing mutation itself, this strategy overcomes important targeting constraints while preserving endogenous *HBB* gene regulation and local genomic architecture. These findings provide proof of concept for precise base editing of mutation-activated cryptic splice sites and establish a foundation for future therapeutic development in β^654^-thalassemia and other inherited disorders caused by aberrant RNA splicing. A schematic summary of this strategy is shown in Fig. 7.

## Discussion

The present study builds directly on our previous nuclease-based studies of β^654^-thalassemia while advancing the therapeutic strategy in an important direction. Our earlier work demonstrated that CRISPR-Cas9-mediated deletion of aberrant splice elements, including the mutation-generated 5′ splice donor site and the activated cryptic 3′ splice acceptor site, can restore β-globin RNA splicing and improve β-thalassemia phenotypes in vivo [17]. Subsequent HDR-mediated modification of the endogenous cryptic 3′ splice site further showed that precise alteration of this splicing element can be sufficient to correct the RNA splicing defect [21]. These studies supported the cryptic splice site as a biologically validated therapeutic target. The current work extends this therapeutic concept by using Td-CBEmax to introduce defined nucleotide substitutions at this validated target without generating DNA double-strand breaks. Compared with nuclease-mediated deletion or HDR-based repair, base editing avoids donor-template dependence, minimizes disruption of the local genomic architecture, and produces more predictable editing outcomes [24, 32, 33]. Thus, the present study represents a progression from target validation to precision, DSB-free modification of a pathogenic splicing element.

A major conceptual advance of this study is the refinement of therapeutic target selection. Conventional genome-editing strategies often focus on directly correcting the disease-causing mutation itself, largely due to PAM accessibility constraints [28, 29]. In contrast, our approach targets the pathogenic molecular event responsible for aberrant RNA processing—the mutation-activated cryptic 3′ splice acceptor site. For the IVS2-654 mutation, activation of this cryptic splice site is indispensable for abnormal β-globin pre-mRNA splicing. By modifying this functionally essential splicing element, normal β-globin pre-mRNA processing was restored without direct correction of the causative IVS2-654 nucleotide substitution. This strategy therefore overcomes the practical limitation imposed by the lack of suitable protospacer adjacent motifs for direct editing of the IVS2-654 mutation while maintaining endogenous *HBB* regulation.

Another important advantage of this strategy is its favorable genome-wide specificity profile. Unlike nuclease-based genome editing, base editing does not rely on DNA double-strand breaks and therefore reduces the potential for large deletions, chromosomal rearrangements, and p53-mediated DNA damage responses reported with nuclease-induced editing [24, 32, 33]. In the present study, targeted deep sequencing together with WGS detected no evidence of widespread off-target editing. Although a small number of candidate variants were identified in one treated mouse, all were located within intergenic regions and were not associated with detectable physiological abnormalities. In addition, systematic analysis of multiple edited genotypes demonstrated that only a subset of nucleotide substitutions effectively restored normal RNA splicing, emphasizing that precise target selection, rather than editing efficiency alone, is critical for successful correction of cryptic splice-site mutations.

The relationship between editing outcome and phenotypic rescue is also important for therapeutic development. In the present study, therapeutic improvement was observed in mice with an allele modification rate of approximately 32%, which is consistent with our previous HDR-based study showing that an editing frequency above approximately 20% can produce therapeutic benefit in β^654^ mice [21]. However, because the current base-editing experiment did not generate a broad series of animals with lower levels of productive editing, the exact minimum threshold required for phenotypic rescue could not be precisely defined. In addition, not all nucleotide substitutions generated within the editing window were functionally equivalent. Some edited genotypes restored normal RNA splicing, whereas others were non-productive and failed to rescue aberrant splicing. Although non-productive edits were not associated with obvious adverse consequences in the present study, bystander editing may be harmful in other sequence contexts. Therefore, future therapeutic development will require optimization of editor selection, sgRNA design, editing-window precision, and the proportion of productive editing outcomes.

Several limitations of the present study should be noted. First, the experiments were performed in a mouse zygotic editing model, which provides a rigorous system for evaluating the biological consequence of defined nucleotide substitutions but does not directly recapitulate the ex vivo HSPC editing and transplantation strategy most relevant for clinical application. Future studies should therefore evaluate this strategy in human hematopoietic stem/progenitor cells and patient-derived cells. Second, although WGS was performed to strengthen the assessment of genome-wide specificity, the sample size remains limited, and extremely rare off-target events cannot be fully excluded. Third, transcriptome-wide analyses were not performed; therefore, potential RNA-level off-target effects or global splicing alterations remain to be evaluated. Finally, the current work focuses on a single β-thalassemia mutation. Whether similar strategies can be applied to additional splicing mutations will require individual evaluation of target accessibility, editing-window compatibility, productive editing outcomes, and safety.

Despite these limitations, the therapeutic concept presented here may be applicable beyond IVS2-654 β-thalassemia. Mutation-activated cryptic splice sites are observed in multiple inherited disorders in which point mutations disrupt normal splice-site recognition or activate aberrant splicing elements. Similar mechanisms are also found in other β-thalassemia-associated splicing mutations, including IVS2-705 and IVS2-745 [34–36]. Although each disease-associated mutation will require individualized evaluation of target accessibility, editing strategy, and functional consequence, our findings support the broader concept that mutation-activated cryptic splice sites can serve as therapeutically actionable targets for precision genome editing. The present study therefore provides a framework for developing DSB-free base-editing strategies for genetic diseases caused by aberrant RNA splicing.

## Conclusion

In summary, this study shows that precise Td-CBEmax-mediated base editing of the mutation-activated cryptic 3′ splice acceptor site restores normal β-globin RNA splicing and substantially rescues disease-associated phenotypes in a β^654^-thalassemia mouse model. Building on previous nuclease-based validation of this pathogenic splicing element, the present work advances cryptic splice-site correction into a defined, DSB-free base-editing framework. By targeting the molecular event responsible for aberrant RNA processing rather than directly correcting the disease-causing mutation itself, this strategy overcomes important targeting constraints while preserving endogenous *HBB* gene regulation and local genomic architecture. These findings provide proof of concept for precise base editing of mutation-activated cryptic splice sites and establish a foundation for future therapeutic development in β^654^-thalassemia and other inherited disorders caused by aberrant RNA splicing.

## Materials and Methods

### Experimental animals

The β^654^-thalassemia mouse model (β^654^ mice) was obtained from The Jackson Laboratory (Bar Harbor, ME, USA). These mice are heterozygous for a human β-globin gene carrying the IVS2-654 splicing mutation, together with a normal murine β-globin allele. Male β^654^ mice were crossed with wild-type (WT) female mice. Embryos at the late one-cell stage were collected and microinjected with either CRISPR-Cas9 components—consisting of single-guide RNA (sgRNA, 50 ng/μL), Cas9 mRNA (100 ng/μL; Invitrogen), and single-stranded oligodeoxynucleotides (ssODNs, 50 ng/μL)—or with the Td-CBEmax system, comprising sgRNA (50 ng/μL) and Td-CBEmax mRNA (100 ng/μL). Injected embryos were transferred into the oviducts of pseudopregnant surrogate females.

Edited mice were categorized as follows:

- β^654−NHDR^: β^654^ mice edited by CRISPR-Cas9 and carried the indel allele near IVS2-579.
- β^654−HDR^: β^654^ mice edited by CRISPR-Cas9 and carried the HDR allele (IVS2-579_581 AA > GG).
- β^654−BE^: β^654^ mice edited by Td-CBEmax and carried base substitutions at the IVS2-579_581 region.

During β^654−NHDR^, β^654−HDR^ and β^654−BE^ founder screening, each germline-edited founder was assessed individually by editing efficiency without random grouping, to independently characterize distinct editing outcomes. For comparisons of WT, β^654^ and β^654−BE^ mice, samples were grouped by genotype and editing status. All animal procedures were approved by the Institutional Animal Care and Use Committee of Shanghai Children’s Hospital and were performed in accordance with the Guide for the Care and Use of Laboratory Animals, with the ethical approval number SHCH-IACUC-2021-XMSB-98 (approval date: 26 October 2021). All animal experiments in this study, including embryo microinjection, animal housing, and phenotyping analysis, were covered under this single ethical approval.

### sgRNA design and screening

The genomic sequence covering 20 bp upstream and 20 bp downstream of the cryptic 3′ splice site IVS2-579 was submitted to the CRISPOR [31] web server for sgRNA design and screening. A total of four candidate sgRNAs were obtained and designated as sgRNA-1, sgRNA-2, sgRNA-3 and sgRNA-4, respectively (Table S1). It has been demonstrated that sgRNAs located closer to the homologous recombination site generally yield higher HDR efficiency. Accordingly, two sgRNAs proximal to the IVS2-579 locus were selected as candidates for CRISPR-Cas9/ssODN-mediated homologous recombination. According to the off-target prediction results generated by CRISPOR, sgRNA-2 harbored more potential off-target sites than sgRNA-1, and the server explicitly warned that this guide sequence had excessive off-target hits and was not recommended for use. Consequently, sgRNA-1 with lower predicted off-target risk was chosen for the generation of gene-edited mice. For the Td-CBEmax base editor, the IVS2-579 site was required to be positioned within the functional editing window. Two candidates, sgRNA-3 and sgRNA-4, met this criterion. Both sgRNAs were separately transfected into HEK293T cells, and the one with higher editing efficiency was selected for subsequent experiments.

### Plasmid construction

U6-sgRNA(sp)-EF1α-GFP plasmids were linearized with BbsI (Thermo Fisher Scientific) and ligated with the corresponding annealed sgRNA oligonucleotide duplexes. Plasmid DNA was purified using the TIANprep Mini Plasmid Kit (TIANGEN Biotech, DP103-03) according to the manufacturer’s instructions.

### Cell culture and transfection

HEK293T cells (Cat. No. SCSP-502) were obtained from the National Collection of Authenticated Cell Cultures (NCACC), Chinese Academy of Sciences, Shanghai. Cells were cultured in DMEM (Gibco) supplemented with 10% (v/v) fetal bovine serum (Sigma) and 1% (v/v) penicillin–streptomycin (Gibco) at 37 °C in a humidified atmosphere containing 5% CO₂. For sgRNA screening, cells were transfected with 750 ng of Td-CBEmax plasmid and 250 ng of sgRNA-3 or sgRNA-4 plasmid using 1.5 μL Lipofectamine 3000 per well. After comparison of editing efficiency, sgRNA-3 was selected for subsequent experiments.

### Genomic DNA extraction

Genomic DNA from cultured cells was isolated by lysis in 150 μL buffer containing 10 mM Tris–HCl (pH 7.0), 0.05% SDS, and 25 μg/mL proteinase K (Thermo Fisher Scientific) at 37 °C for 1 h, followed by heat inactivation at 80 °C for 30 min. Genomic DNA from mouse tail biopsies was extracted using the One Step Mouse Genotyping Kit (Vazyme) according to the manufacturer’s instructions.

### Off-target prediction and targeted deep sequencing validation

Potential off-target loci of sgRNA-3 were computationally predicted using the webtool CRISPOR v5.2 (http://crispor.tefor.net) [31]. Briefly, the full sgRNA sequence together with canonical NGG PAM was submitted to the pipeline, with human reference genome hg19 selected as the background. The CFD scoring model was adopted for off-risk evaluation, and candidate loci carrying ≤ 4 mismatches against sgRNA were retained and ranked by CFD off-target risk score. The top 10 high-risk predicted off-target loci with the highest CFD scores were selected for subsequent targeted deep sequencing validation (Table S2). Chromosomal position, mismatch pattern and gene name of each candidate site were exported from CRISPOR. Human HEK293T cells were divided into two groups: editing group transfected with sgRNA-3 and base editor expression plasmid, and untransfected blank control group; three independent biological replicates were set for each group. Seventy-two hours post-transfection, cell pellets were collected and genomic DNA was extracted using a commercial genomic DNA extraction kit. Flanking sequences covering about 100 bp upstream and downstream of each on-target and predicted off-target locus were amplified by KOD high-fidelity DNA polymerase (TOYOBO). Detailed forward and reverse primer sequences for all amplicons are summarized in Table S1. PCR products were separated via agarose gel electrophoresis and purified with QIAquick Gel Extraction Kit (Qiagen). Purified fragments were subjected to end repair, A-tailing and Illumina adapter ligation to construct sequencing libraries. Library concentration was quantified by Qubit fluorometer, and libraries were sequenced on Illumina NextSeq 500 platform (2 × 150 bp paired-end reads), with a minimum of 10,000 high-quality filtered reads acquired per amplicon. Sequencing data were analyzed using CRISPResso2 software [37]. One tailed Student’s t tests were used to compare mean editing frequencies between edited and control (unedited) samples for each target site.

### Whole-genome sequencing and data analysis in HEK293T cells

Edited and control cell samples were subjected to 30 × average-depth WGS on the MGI-T7 platform. Raw reads were demultiplexed and aligned to hg38 via BWA-MEM v0.7.7, with downstream processing as previously described [38]. Sorted deduplicated BAMs underwent GATK v3.4 base recalibration; sequencing depth and coverage were calculated using mosdepth v0.2.6 and bedtools genomecov. Variants were independently called with GATK HaplotypeCaller v4.1.3.0, FreeBayes v1.3.1 and VarScan v2.4.3. Raw variants failing thresholds QD < 2, FS > 60, SOR > 3, MQ < 40, MQRankSum < − 5 were discarded. bcftools v1.9 removed variants present in controls, and only variants detected by all three tools were retained as high-confidence sites. Before WGS, Cas-OFFinder [39] predicted sgRNA off-target candidates, which were overlapped with filtered WGS variants. Candidates were classified through semi-local alignment of ± 30 bp flanking sequences against the sgRNA: loci with ≤ 6 mismatches (no bulge) or ≤ 3 mismatches (one bulge) were sgRNA-dependent off-targets, others sgRNA-independent. Candidates with extreme GC (< 25%/> 75%), homopolymers or repetitive localization were excluded to eliminate false positives. All globin homologous genes and adjacent regulatory regions were manually curated to assess functional-region off-target editing.

### Whole-genome sequencing and data analysis in β^654−BE^ mice

Splenic gDNA from six Td-CBEmax-edited β^654−BE^ mice and four untreated β^654^ controls (2 males, 2 females) was subjected to WGS on the MGI-T7 platform at 90 × (edited) and 50 × (control) average depth. Clean reads were mapped to mouse reference genome GRCm39 (Ensembl release 110), following the identical cellular WGS pipeline above: alignment, deduplication and base recalibration. SNVs and small indels were called via Samtools, vcftools and picard-tools, retaining only high-quality variants after uniform QC filtering. Germline variants shared with controls were subtracted to extract editing-specific mutations per edited mouse. For off-target screening, Cas-OFFinder predicted sgRNA-homologous loci with ≤ 5 mismatches (no bulge). Overlaps between predicted sites and editing-unique variants were defined as sgRNA-dependent off-targets. Candidates were filtered by the same GC/homopolymer/repeat exclusion criteria mentioned above. All globin-family genes and their regulatory regions were manually inspected to rule out functional hazardous off-target edits.

### In vitro transcription of sgRNA and Td-CBEmax mRNA

DNA templates were generated by PCR using pX459-sgRNA plasmids and specific primers (Table S1). sgRNAs were transcribed in vitro using the HiScribe™ T7 Quick High Yield RNA Synthesis Kit (New England Biolabs) and purified with the MEGAclear Kit (Life Technologies). Td-CBEmax plasmids were linearized with BspQI, purified, and transcribed using the HiScribe® T7 ARCA mRNA Kit (New England Biolabs).

### Generation of gene-edited β^654^ mice

We first generated HDR-modified β^654^ mice by microinjecting sgRNA-1, Cas9 mRNA, and ssODN into fertilized zygotes to verify that defined base substitutions at the cryptic 3′ splice acceptor site could rescue the abnormal splicing caused by the β^654^ mutation. We then generated base-edited β^654^ mice by microinjecting sgRNA-3 and Td-CBEmax mRNA into fertilized zygotes. Gene-edited offspring were genotyped at approximately three weeks of age. Survival rates were calculated based on the expected Mendelian ratio of β^654^ to WT offspring.

#### RT-PCR analysis

Total RNA was reverse transcribed using the HiScript® II Reverse Transcriptase Kit (Vazyme). β-globin transcripts were amplified using IVS2-654-specific primers, yielding a 399 bp product for correctly spliced transcripts and a 472 bp product for aberrantly spliced transcripts. Mouse *Gapdh* served as an internal control.

#### HPLC

For high-performance liquid chromatography (HPLC) measurement, 50 μL whole blood was harvested, diluted with 1 mL ultrapure water and filtered to obtain test samples. Subsequently, 10 μL prepared sample was injected onto a ChromCore 300 C4 reversed-phase polypeptide column (300 Å, 5 μm, 4.6 mm × 250 mm). Quantification of distinct globin chains was performed using an Agilent 1260 liquid chromatography system. Mobile phase consisted of water and acetonitrile supplemented with 0.1% trifluoroacetic acid, running with a linear gradient ranging from 40 to 80% at a flow rate of 1 mL/min.

#### Hematologic analysis

For morphological evaluation of blood cells, 1–2 μL peripheral blood obtained from mouse tail tips was utilized for the preparation of blood smears, followed by Wright-Giemsa staining (Baso, Zhuhai, China). For quantitative hematological profiling, peripheral blood specimens from mice were harvested using heparin-pretreated microhematocrit tubes. The measurement of hematological parameters was performed on a Sysmex XT-2000iV hematology analyzer (Japan). The detected indices covered red blood cell counts, hemoglobin concentrations, hematocrit, mean corpuscular volume, mean corpuscular hemoglobin, mean corpuscular hemoglobin concentration and reticulocyte percentage.

#### Histopathology analysis

Blood and bone marrow smears were stained with Wright-Giemsa (Baso, Zhuhai, China). Liver and spleen tissues from WT, β^654^, and β^654−BE^ mice were embedded in paraffin, sliced into 4 µm sections, and stained with hematoxylin and eosin (Baso, Zhuhai, China) and Prussian Blue (Baso, Zhuhai, China).

#### Statistical analysis

Statistical analyses were performed using GraphPad Prism 9. Data are presented as mean ± standard deviation. Group comparisons were conducted using one-way ANOVA or multiple non-parametric t-tests with Holm–Šídák correction. A *P* value < 0.05 was considered statistically significant.

## Supplementary Information

Supplementary Material 1.

Supplementary Material 2.

Supplementary Material 3.

## Data Availability

The data supporting the findings of this study are available from the corresponding author upon reasonable request. Whole-genome sequencing data have been deposited in the Genome Sequence Archive (GSA) at the National Genomics Data Center (CNCB-NGDC) under accession number CRA047856.
