# Supplementary material for: Precision base-editing of the cryptic 3’ acceptor site to correct the RNA splicing defect of β654-thalassemia
Source: Mol Biomed. 2026 Sep 30;7:190. doi: 10.1186/s43556-026-00589-z (PMC13627643; doi:10.1186/s43556-026-00589-z)
Supplement: Supplementary file 1 [file 43556_2026_589_MOESM1_ESM.docx]

**Precision Base-Editing of the Cryptic 3’ Acceptor Site to Correct the RNA Splicing Defect of β^654^-thalassemia**

Dan Lu,^1,2,3*^ Xiuli Gong,^1,3^ Xinbing Guo,^1,3^ Qin Cai,^1,3^ Wenxiu Li,^1,3^ Yanwen Chen,^1,3^ Dali Li^1,3,4*^, and Fanyi Zeng^1,2,3,5*^

1 Shanghai Institute of Medical Genetics, Shanghai Children's Hospital, Shanghai Jiao Tong University School of Medicine, Shanghai, 200040, China

2 Department of Histo-Embryology, Genetics and Developmental Biology, Shanghai Jiao Tong University School of Medicine, Shanghai, 200025, China

3 NHC Key Laboratory of Medical Embryogenesis and Developmental Molecular Biology & Shanghai Key Laboratory of Embryo and Reproduction Engineering, Shanghai, 200040, China

4 Shanghai Frontiers Science Center of Genome Editing and Cell Therapy, Shanghai Key Laboratory of Regulatory Biology, Institute of Biomedical Sciences and School of Life Sciences, East China Normal University, Shanghai, 200241, China

5 School of Pharmacy, Macau University of Science and Technology, Macau, China

*Correspondence:

Fanyi Zeng, M.D., Ph.D.

E-mail: fzeng@vip.163.com

Dali Li, Ph.D.

E-mail: dlli@bio.ecnu.edu.cn

Dan Lu, Ph.D.

E-mail: danlu125@163.com

Running heads: Base editor therapy for β^654^-thalassemia

**Online Supplementary Data**

**Table S1. Primer sequences.**

| **Primer name** | **Primer sequence** | **Utility** |
| --- | --- | --- |
| sgRNA-1 | TCCCTAATCTCTTTCTTTCA | Guide sequences |
| sgRNA-2 | TTCCCTAATCTCTTTCTTTC |  |
| sgRNA-3 | TGCCCTGAAAGAAAGAGATT |  |
| sgRNA-4 | GCCCTGAAAGAAAGAGATTA |  |
| T7-sgRNA-3 | TAATACGACTCACTATAGGTGCCCTGAAAGAAAGAGATT | Preparation of sgRNAs |
| T7-sgRNA-1 | TAATACGACTCACTATAGGTCCCTAATCTCTTTCTTTCA |  |
| G-R | AAAAAAGCACCGACTCGGTG |  |
| Mhbb-QF1 | TGGGCAGGCTGCTGGTTGTC | qPCR for copy number of mouse β-major globin gene |
| Mhbb-QR1 | CAAGTGATTCAGGCCATCGTT |  |
| Mus TF-F | TGACTGCACCGGCAATTTC | qPCR for copy number of mouse transferrin gene |
| Mus TF-R | GGTACCCTCTGGAAGTTTAACGAA |  |
| β-L | GACCAAATCACGGTAATTTTGC | PCR detection of the human β-globin gene (616 bp) |
| β-R | GGCAGAATCCAGATGCTCAA |  |
| β-L-READ1 | TCGTCGGCAGCGTCAGATGTGTATAAGAGACAGGACCAAATCAGGGTAATTTTGC | Primers to amplify the Endogenous target site for amplicon deep sequencing |
| R2-READ2 | GTCTCGTGGGCTCGGAGATGTGTATAAGAGACAGGAATGGTGCAAAGAGGCATGA |  |
| OT1-F | TCGTCGGCAGCGTCAGATGTGTATAAGAGACAGATGAATTCATGCCAAA | Primers to amplify the predicted off-target sites for amplicon deep sequencing |
| OT1-R | GTCTCGTGGGCTCGGAGATGTGTATAAGAGACAGATTGCTCTGGAATTAGAAGTT |  |
| OT2-F | TCGTCGGCAGCGTCAGATGTGTATAAGAGACAGAGATTAAGATGCCCATAAGA |  |
| OT2-R | GTCTCGTGGGCTCGGAGATGTGTATAAGAGACAGTAGCCAAGAAGGAGGGATATT |  |
| OT3-F | TCGTCGGCAGCGTCAGATGTGTATAAGAGACAGGGGGAAGAGAATTTGACTGA |  |
| OT3-R | GTCTCGTGGGCTCGGAGATGTGTATAAGAGACAGGGTGTCAAAATGTATCAAACAGTA |  |
| OT4-F | TCGTCGGCAGCGTCAGATGTGTATAAGAGACAGTTTGGGTTAAATCTGCTTAGT |  |
| OT4-R | GTCTCGTGGGCTCGGAGATGTGTATAAGAGACAGCAATTGGCGTACTGAT |  |
| OT5-F | TCGTCGGCAGCGTCAGATGTGTATAAGAGACAGTTTTGTTTATCTGGAACTGTC |  |
| OT5-R | GTCTCGTGGGCTCGGAGATGTGTATAAGAGACAGAGCAGGAGAGAAGTAACTC |  |
| OT6-F | TCGTCGGCAGCGTCAGATGTGTATAAGAGACAGAGTCCAGGAGGTTGAGG |  |
| OT6-R | GTCTCGTGGGCTCGGAGATGTGTATAAGAGACAGATGAGATGAGCCAGTGATAGA |  |
| OT7-F | TCGTCGGCAGCGTCAGATGTGTATAAGAGACAGATGAAGGCATTGATGGATATA |  |
| OT7-R | GTCTCGTGGGCTCGGAGATGTGTATAAGAGACAGGGGGACTAGCAAGTCAG |  |
| OT8-F | TCGTCGGCAGCGTCAGATGTGTATAAGAGACAGGGCAGTGGTCAGGCCTTAAT |  |
| OT8-R | GTCTCGTGGGCTCGGAGATGTGTATAAGAGACAGCCACAGACATGGCGTAACCTT |  |
| OT9-F | TCGTCGGCAGCGTCAGATGTGTATAAGAGACAGACACTCCACACCTTTGTTCCA |  |
| OT9-R | GTCTCGTGGGCTCGGAGATGTGTATAAGAGACAGAGTCTAGGCAGGTAGTCAGCTG |  |
| OT10-F | TCGTCGGCAGCGTCAGATGTGTATAAGAGACAGATACGAGGATGACCATATGTA |  |
| OT10-R | GTCTCGTGGGCTCGGAGATGTGTATAAGAGACAGAAAACTTAAGAACACGCTA |  |
| HGB-L | GAGGAGAAGTCTGCCGTTAC | RT-PCR for human β-globin (399 bp or 472 bp) |
| HGB-R | AGCCACACCAGCCACCACTT |  |
| GAPDH-L | AGGCCGGTGCTGAGTATGTC | RT-PCR for mouse *Gapdh*(530 bp) |
| GAPDH-R | TGCCTGCTTCACCACCTTCT |  |
| Q-Gapdh-F | AGGTCGGTGTGAACGGATTTG | qPCR for mRNA expression of mouse *Gapdh* gene |
| Q-Gapdh-R | GGGGTCGTTGATGGCAACA |  |

**Table S2. Summary of predicted off-target sites identified by CRISPOR**

| **Site number** | **Location** | **Gene name** | **Sequences** |
| --- | --- | --- | --- |
| On Target | chr11_5225995-5225975:- | *HBB* | TGCCCTGAAAGAAAGAGATT |
| OT1 | chr3:174204131-174204153:- | *RN7SKP234-NLGN1* | TGCTCCAAAAGAAAAAGATT |
| OT2 | chr12:93077796-93077818:+ | *Y_RNA-RP11-202G11.2* | GGACCAGAAAGAAAGAAATT |
| OT3 | chr1:245015063-245015085:- | *EFCAB2* | CACCCAGAAAGAAAGAGATT |
| OT4 | chr14:52992879-52992901:- | *FERMT2-DDHD1/RP11-368P15.3* | AGCCTCAAAAGAAAGAGATT |
| OT5 | chr11:113667535-113667557:- | *DRD2-TMPRSS5* | AATCCTGAAAGAAAAAGATT |
| OT6 | chr5:149321993-149322015:+ | *AFAP1L1* | TGCACTCAAAAAAAGAAATT |
| OT7 | chr11:105272413-105272435:+ | *RP11-94P11.4-Metazoa_SRP* | TTCCCCAAAAGAAAGAAATT |
| OT8 | chr12:62532287-62532309:+ | *MON2* | TTCCCTAAATGAAAGAAATT |
| OT9 | chr4:170395548-170395570:+ | *RP11-789C1.2-RP11-322J23.1* | TCTCCTGAAAGAAAGAGATT |
| OT10 | chr13:42045543-42045565:- | *RP11-187A9.3-DGKH* | TTACCTGAATGAAAAAGATT |

The red letters indicate mismatched bases relative to the on-target sequence.

**Table S3 Base-editing sites and status of splicing pathway**

| Base-editing Site | Sequences after base-editing（IVS2-578 to IVS2-581） | Status of splicing pathway |
| --- | --- | --- |
| β^654^ | AGGG | aberrant |
| IVS2-579 G>A | AAGG | correct |
| IVS2-579 G>T | ATGG | correct |
| IVS2-579 G>C | ACGG | correct |
| IVS2-580 G>A | AGAG | aberrant |
| IVS2-581 G>A | AGGA | aberrant |
| IVS2-579_580 GG>AA | AAAG | correct |
| IVS2-579_580 GG>TA | ATAG | aberrant |
| IVS2-580_581 GG>TA | AGTA | aberrant |

**Table S4. Hematologic analyses of F0 and F1 β^654-BE^ mice.**

| Group | N | RBC (10^12^/L) | HGB (g/L) | HCT (%) | MCV (fL) | MCH (pg) | RET (%) |
| --- | --- | --- | --- | --- | --- | --- | --- |
| WT | 8 | 9.5±0.3 ^*^ | 144.1±8.3 ^*^ | 47.2±2.7 ^*^ | 49.6±2.8 ^*^ | 15.1±0.9 ^*^ | 3.1±0.5^*^ |
| β^654-BE^ | 6 | 9.8±0.5 ^*^ | 151.3±9.5 ^*^ | 50.3±3.3 ^*^ | 51.2±1.4 ^*^ | 15.4±0.7 ^*^ | 4.7±2.2^*^ |
| F1-β^654-BE^ | 12 | 9.9±1.1 ^*^ | 152.9±16.6 ^*^ | 51.6±4.8 ^*^ | 52.4±1.8 ^*^ | 15.5±0.4 ^*^ | 4.0±0.8 ^*^ |
| β^654^ | 11 | 6.5±1.0 | 84.6±7.7 | 29.4±4.3 | 45.1±3.9 | 13.1±1.7 | 26.2±8.1 |

A One-Way ANOVA test was used for intergroup comparisons. Values represent mean ± standard deviation. Statistically significant differences are indicated for WT, β^654-BE^ or F1-β^654-BE^ compared with β^654^. * P<0.01. N: number of mice tested; RBC: red blood cell count; HGB: hemoglobin; HCT: hematocrit; MCV: mean corpuscular volume; MCH: mean corpuscular hemoglobin; RET: reticulocyte count.

**Table S5. Initial and filtered unique variant counts from whole-genome sequencing.**

| Group | Sample No. | Counts of Indel | Unique Indel | Counts of SNV | Unique SNV |
| --- | --- | --- | --- | --- | --- |
| β^654^ | 1 | 429875 | - | 1272965 | - |
|  | 2 | 401426 | - | 1225484 | - |
|  | 3 | 324740 | - | 838206 | - |
|  | 4 | 364963 | - | 1127096 | - |
| β^654-BE^ | 1 | 452140 | 50493 | 1109138 | 118513 |
|  | 2 | 386530 | 28609 | 785120 | 27077 |
|  | 3 | 459806 | 55595 | 1178977 | 154279 |
|  | 4 | 430721 | 27789 | 1102099 | 12731 |
|  | 5 | 378361 | 24681 | 806857 | 11411 |
|  | 6 | 414075 | 31016 | 1009492 | 52508 |

**Figure S1. Schematic of sgRNA-4 target site and editing activity detected in HEK293T cells**

(a) Schematic of sgRNA-4 target site. The sgRNA-4 binding sequence is underlined, while nucleotides within the predicted editing window spanning IVS2-579_581 GGG are labeled; protospacer adjacent motif (PAM) sequences are highlighted..
(b) Nucleotide distribution surrounding the sgRNA-4 target site following Td-CBEmax editing.
(c) Distribution of edited alleles at the target locus as determined by deep sequencing. Nucleotides are color-coded (A, green; C, red; G, yellow; T, purple). Substituted bases are shown in bold.

**Figure S2. Targeted deep sequencing of amplicons of the top ten predicted off-target sites.**

Using the CRISPOR tool, 10 potential genomic off-target sites with 4 or fewer mismatches to the on-target sequence were identified. Each site was evaluated by amplicon deep sequencing. Dot graphs show the percentage of sequencing reads containing C•G-to-T•A conversions within protospacer positions 1–11 at off-target sites in genomic DNA samples from HEK293T cells treated with Td-CBEmax plasmid, or untreated controls (n = 3). * indicates off-target sites with significant difference in edit frequency between mock and edited samples (*P<0.05; ** P<0.01). Each input sample is from an independent experiment.

**Figure S3. Genotyping of β^654-BE^ founder mice using Sanger sequencing**

Representative Sanger sequencing chromatograms of β^654^ mice and β^654-BE^ founder mice (βBE3–7) injected with Td-CBEmax mRNA and sgRNA-3.

**Figure S4. RT-PCR to Identify normal and aberrant β-globin splicing products using Sanger sequencing validation.**

(a) Representative Sanger sequencing chromatograms of the 399-bp product, corresponding to normally spliced β-globin transcripts, amplified from founder mouse βBE4.

(b) Representative Sanger sequencing chromatograms of the 472-bp product, corresponding to aberrantly spliced β-globin transcripts, amplified from founder mouse βBE7.

(c) Sequence alignment of the 472-bp and 399-bp RT-PCR products, confirming the presence of the abnormally inserted exon sequence in the longer fragment.

**Figure S5. Expression analysis of β-globin gene in β^654-BE^ mice.**

Representative high-performance liquid chromatography chromatograms. In β^654^ mice, peaks corresponding to mouse α-globin and mouse β-major globin are shown. In β^654-BE^ mice, peaks corresponding to human β-globin, mouse α-globin, and mouse β-major globin are indicated. Red arrows denote human β-globin peaks.

**Figure S6. Genome-wide SNV and small indel loads in Td-CBEmax-edited β^654-BE^ mice versus untreated β^654^ controls.**

(a) Bar plots overlaid with individual scatter points showing the average number of genome-wide SNVs for β^654-BE^ (n = 6) and control β^654^ mice (n = 4).

(b) Bar plots with overlaid dot graphs depicting the mean counts of small insertions and deletions (Indels) in the two mouse groups. No statistically significant differences in SNV or indel abundance were detected between the edited and control cohorts. Raw quantitative data are provided in Table S4.
